# Immunoregulatory impact of human mesenchymal-conditioned media and mesenchymal derived exosomes on monocytes

**DOI:** 10.22099/mbrc.2019.33346.1397

**Published:** 2019-06

**Authors:** Samaneh Tokhanbigli, Kaveh Baghaei, Ali Asadirad, Seyed Mahmoud Hashemi, Hamid Asadzadeh-Aghdaei, Mohammad Reza Zali

**Affiliations:** 1 Basic and Molecular Epidemiology of Gastrointestinal Disorders Research Center, Research Institute for Gastroenterology and Liver Diseases, Shahid Beheshti University of Medical Sciences, Tehran, Iran; 2 Department of Immunology, School of Medicine, Shahid Beheshti University of Medical Sciences, Tehran, Iran; 3 Gastroenterology and Liver Diseases Research center, Research institute for Gastroenterology and Liver Diseases, Shahid Beheshti University of Medical Sciences, Tehran, Iran

**Keywords:** Mesenchymal stromal cells, Conditioned media, Exosomes, Pro-inflammatory, anti-inflammatory cytokines

## Abstract

Mesenchymal stem cells (MSCs) are well known due to their immunomodulatory effect, but the exact mechanisms have not been defined. Several studies demonstrated that the exerted immunoregulatory effect of these cells could be mediated by paracrine factors to illustrate, cytokines, chemokine, and among which, extracellular vesicles are one of them to play a crucial role. Moreover, it is assumed that extracellular vesicles are an essential player in intracellular communication by transferring their component. In this respect, the efficiency of conditioned media and exosomes was compared to illustrate a practical approach to cell-free based therapies. In the current study, we investigated the effect of both MSCs conditioned media (MSC-CM) and MSCs-derived exosomes on the expression of pro-inflammatory and anti-inflammatory cytokines in peripheral blood mononuclear cells (PBMCs). In this regard, isolated PBMCs were treated with MSC-CM and MSC-derived exosome as separated groups. Expression of inflammatory and anti-inflammatory markers was evaluated by Real-time PCR and ELISA. The immunoregulatory effect of MSC-CM on pro-inflammatory and anti-inflammatory genes, such as IL-12b, iNOS, EGR-2, IL-10 with an exception in case of IL-6 was more significant. Whereas in protein levels IL-10 showed the most substantial difference in exosome treated groups. It could be assumed that MSC-CM has more immunoregulatory impact on monocyte in contrast with exosomes.Taken together, by considering the recent approaches to cell-free therapy and the immunoregulatory impact of MSCs, yet relatively little is known about the efficacy of human-MSC-CM and secreted exosome compared with each other.

## INTRODUCTION

In terms of multipotency, Mesenchymal Stromal Cells (MSCs) are considered as adult stem cells with self-renewal and differentiation features. These cells are present in many tissues such as bone marrow [1], adipocytes [2, 3], umbilical cord blood [4], tooth pulp [5, 6] and, peripheral blood [7] with differentiation capability to chondrocytes, osteoblasts and adipocyte (mesodermal lineage) [8, 9], neuron (ectodermal lineage) [10], and hepatocyte (endodermal lineage) [11]. These cells exert immunoregulatory effects by either cell to cell contacts or secreting biologically active substances. These functions are performed mostly by suppressing B-cells, NK cells and T-cells proliferation, and even has an inhibitory impact on the differentiation of Dendritic Cells [12, 13]. Therefore, MSCs are represented as one of the well-known cell sources in cellular immune therapies for a variety of inflammatory associated and autoimmunity diseases such as Crohn’s disease, ulcerative colitis [14, 15], multiple sclerosis [16], and systemic lupus erythematosus [17, 18]. 

MSCs secret arrays of factors such as cytokines, soluble mediators, and exosomes into their microenvironment, which are responsible for the dual role of immunomodulatory, inflammatory, and anti-inflammatory aspects of these cells. Macrophage colony-stimulating factor (M-CSF), interleukin-6 (IL-6), IL-11, IL-15, stem cell factor, and vascular endothelial growth factor are parts of secreted factors. These cytokines are involved in hematopoiesis regulation, cell signaling, and modulation of the immune responses. Hence, these distinct roles of MSCs and secreted cytokine panel open a tremendous perspective in regenerative medicine and cell therapy   [19, 20] . 

Despite large scale MSC therapy in recent years, the limitations remain due to their expansion in vitro to reach reasonable amount to inject and even their finite capacity of passage numbers. Therefore, MSCs conditioned media are proposed as an alternative which has been used to treat kidney injury to myocardial damage in mouse models   [21, 22] . In this context, another emerging era in cell free therapeutic approaches is using MSC derived extracellular vesicles (MSC-EVs) as part of cell secretion which are known as MSc derived exosomes. Exosomes are the reflection of the biology of their origin cell containing proteins, RNAs, and lipids   [23]  involved in various cellular activities and physiological processes. Besides, these secreted exosomes from non-immune and immune cells have important roles in immune regulation and in MSC case it has been hypothesized that the immunomodulatory effect of MSCs could be carried out through these exosomes. Moreover, extracellular vesicle-based therapy specifically exosomes are being established as cell-free therapeutic agents in clinical for the treatment of inflammatory diseases, autoimmune disorders, and cancer in recent years.

On the other hand, Substantiate with evidence, the interaction of MSCs with monocytes could play a significant role in their immune modulatory effects of these subjected cells. Monocytes are a population of PBMCs, which in response to their microenvironment they can be differentiated to various populations namely macrophages. The plasticity of these cells and occurrence of phenotype changes is mostly through their environmental signals. M1 macrophages are considered to encourage inflammation, whereas M2 has a specific role in the decline of inflammation and support the wound healing and tissue repair process   [24, 25] . Overall, in the present study, we investigated the immunregulatory of both MSCs conditioned media and exosome’s influence on monocytes and occurred changes in their cytokine expression.

## MATERIALS AND METHODS

**Isolation, purification, and cultivation of bone marrow derived mesenchymal stem cells (MSCs): **Human bone marrow (BM) was collected from the patient’s iliac crest treating for transplantation in the Taleghani Hospital after having their written consent according to Shahid Beheshti University of Medical Sciences (SBMU) and the Research Institute for Gastroenterology and Liver Diseases (RIGLD) ethical committee, code of ethics (IR.SBMU.RIGLD.REC.1395.123). Collected BM aseptically was transferred to K_2_EDTA tube. The buffy coat was isolated by centrifugation (450 × *g*, 10 min). The separated buffy coat was layered onto an equal volume of Ficoll (GE health care, USA) and centrifuged (400 × *g*, 20 min). Cells at the interface were removed and washed twice in sterile PBS. 

Prior to conducting the research, current project’s protocol was reviewed and approved by the Shahid Beheshti University of Medical Sciences (SBMU) and the Research Institute for Gastroenterology and Liver Diseases (RIGLD) ethical committee.

Human bone marrow progenitor cells were cultured on tissue treated culture plates in Dulbecco's Modified Eagle's medium (DMEM) supplemented with 10% FBS and penicillin/streptomycin (50 U/ml and 50 mg/ml, Gibco-Invitrogen, Carlsbad, USA; respectively). Plates were maintained at 37°C in a humidified atmosphere containing 5% CO_2 _for 48h. In order to remove the non-adhered medium exchange carried out. The cultures were maintained for an additional one week with a medium exchange. 

**Assessing the differentiation quality of bone marrow derived mesenchymal stem cells (MSCs): **Osteoblastic and adipocyte differentiation was assessed by culturing 60% confluent human MSCs for 21 days in differentiation media (all from Sigma). Medium exchange was done every 3 days and at the last day of differentiation, cells were stained with Alizarin (PromoCell) to detect calcium-rich nodules and intracellular Oil-Red-O (PromoCell) for detection of fat droplets. 

**Flow cytometric analysis and characterizing of human mesenchymal stem cells (hMSCs): **To characterize the adherent cells, both differentiation assay and flow cytometry analysis were carried out. Flow cytometry analysis was used to assess the immune profile of MSC cells, using the standard described by the International Society for Cellular Therapy (ISCT) for MSC. Cells (P2-3) were harvested, and suspended in 2% bovine serum albumin (BSA in PBS), and counted. From each population, 10^5^ cells were used for flow cytometry. Cells directly were stained with PE (phycoerythrin) conjugated antibodies against CD14, CD34, CD45, CD90, CD105 and CD73 (ebioscience, Germany). An appropriate isotype-matched control antibody (mouse IgG1 K Iso control) was used in all analysis. Cells were analyzed by FACS flow cytometry using Cell Quest Software (Becton Dickinson, UK). 

**MSCs conditioned media:** Isolated Mesenchymal Stem Cells from human bone marrow were cultivated in Dulbecco's modified Eagle's medium (DMEM) (Gibco; Thermo Fisher Scientific, Inc., Waltham, MA, USA) supplemented with 10% fetal bovine serum (FBS) penicillin/streptomycin (50 U/ml and 50 mg/ml, Gibco-Invitrogen, Carlsbad, USA; respectively). Every 2-3 days the medium was changed and when the cells reach 80% confluency the percentage of FBS was being reduced to 10%, 5%, and ultimately 2% every 3 days. Subsequently, the 2%, conditioned media was collected after 5 days in order to treat the monocytes.

**Isolation and identification of MSC derived exosome:** At 80% confluency, cultivated human bone marrow derived MSCs medium was exchanged to serum-free medium (complete medium containing no FBS). After 5 days, the medium was collected and centrifuged at 16,000 ×g for 30 minutes to remove any remaining cell debris. After centrifuging, ½ volume of Exo-spin™ Buffer was added, mixed well and incubated at 4°C for at least 1 hour or overnight. The mixture was centrifuged at 16,000×g for 1hour and finally, the supernatant was discarded. The exosome containing plate was applied to the Exo-spin™ column according to manufacturer’s protocol. 

**Characterization of exosomes:** The size, concentration, morphology of isolated exosomes was identified by Dynamic light scattering (DLS) (Malvern Instruments Ltd, Worcestershire, UK.), BCA (Bicinchoninic Acid) Protein Assay kit (Thermo Fisher Scientific, Rockford, USA) and Scanning Electron Microscope (SEM) (KYKY-EM3200, China).

**Monocyte isolation and cultivation:** Human PBMCs from healthy donor (Written consent was provided prior to participation) collected in K_2_EDTA tube. PBMCs were diluted 1:1 with Dulbecco's Phosphate Buffered Saline (D–PBS) (Life technologies, USA) without Mg^2+^ and Ca^2+^. PBMCs were isolated using gradient centrifuge by layering on top of ficoll (GE health care, USA), and collected PBMCs were washed twice in PBS and applied to negative sorting using the monocyte isolation Kit II (Miltenyi Biotec, Bergisch Gladbach; Germany) according to manufacturer’s protocol. Isolated monocytes were cultured in RPMI, supplemented with 10% FBS and penicillin/streptomycin. 

**Human monocyte confirmation by flow cytometry: **Isolated monocytes from Human PBMCs were taken to flow cytometry analysis to confirm the purity of the CD14 positive monocytes population as it was described before. 

**Co-culture of** **monocytes with** **MSCs-derived exosomes and** **MSC-derived conditioned media:** 24 hours after monocyte culture in defined medium, complete medium (10% FBS) was replaced with MSC derived conditioned media and complete medium in 7:3 ratio respectively. The other test flask was treated with 50μg/ml MSC-derived exosome. The control cells were cultured in complete RPMI medium without any treatment. Both tests and control flasks were incubated for 72 hours at 37^0^C, with 5% CO2, and 90% humidity.

**Characterization of CD45 population by flow cytometry analysis:** 72 hours after treatment, flow cytometry was performed in co-cultured cells to assess CD45 surface marker common among monocyte and macrophage population by determining the CD45 positive cells in compared with the control group.

**RNA extraction, cDNA synthesis, and quantitative reverse transcription PCR (RT-qPCR):** After 72 hours, RNAs of tests and control cells were extracted by YTA Total RNA Purification Mini Kit (FavorGen, Taiwan) according to manufacturer’s protocol. Extracted total RNA was used in the next step for cDNA synthesis using random hexamers in the presence of RNase inhibitor (RevertAid First Strand cDNA Synthesis Kit, cat no K1622, Thermo Scientific**).** Synthesized cDNA was applied to Real-time quantitative PCR assays. Real-Time was performed on Rotor Gene Q Series Real-Time PCR system thermal cycler and SYBR Green Mastermix (Applied Biosystems). Obtained data were normalized by GAPDH (used as a reference gene) in analysis process. Primers used to evaluate pro-inflammatory and anti-inflammatory cytokine expression levels were designed by primer3, NCBI, the sequence of IL-6 F: 5΄-TACATCCTCGACGGCATCTC-3΄ R: 5΄-AGTGCCTCTTTGCTGCTTTC-3΄, IL-12b F: 5΄-AAGAATTCTCGGCAGGTGG-3΄ R: 5΄-ACGCAGAATGTCAGGGAG-3΄, iNOS F: 5΄-CCCAAGCTCTACACCTCC -3΄ R: 5΄-AACACGTTCTTGGCATGC-3΄, EGR-2 F: 5΄-CATTGGGAAGAGACCTGGG -3΄ R: 5΄-ACCTCCACCTCTTCCTCTC-3΄, and IL-10 F: 5΄-TTCCATTCCAAGCCTGACCA -3΄ R: 5΄-ATTTGTAGCAGTTAGGAAGCCC-3΄ are accordingly. 

**Enzyme-linked immunosorbent assay (ELISA):** Concentration of IL-10 in both groups was quantified by an ELISA kit (R&D Systems) as follows. In the first step, conditioned media after 72h from control, conditioned media treated, and exosome treated groups were collected. In the next step, experiments were performed according to the manufacturer’s instructions. After performing all treatments and incubations, colorimetric absorbance was recorded at a wavelength of 450 nm.

**Statistics:** Collected data were analyzed and the changes in mRNA expression were compared with control group with relative expression levels of targeted mRNA over the reference values. Collected data were analyzed by the GRAPH PAD PRISM5.0 software using one-way analysis of variance ANOVA method. P<0.01 was considered statistically significant.

## RESULTS

Based on our previous published study   [26]  human MSCs isolation was carried out by Ficoll-Paque. After isolation, cultivated cells maintained in defined media. After 8–12 days, the majority of non-adherent cells were removed during the medium exchanges. The remaining cells had a heterogeneous spindle shaped morphology and exhibited distinct colony formation. The hMSC cultures had significant proliferation, which gradually resulted in the maintenance of a homogeneous fibroblastic morphology. The cultivation of MSCs under adipogenic differentiation medium for 21 days proved the positive lipid droplets in the majority of cells and calcium oxalates formation in the differentiated MSCs by Alizarin staining (Data not shown).

**Figure 1:**
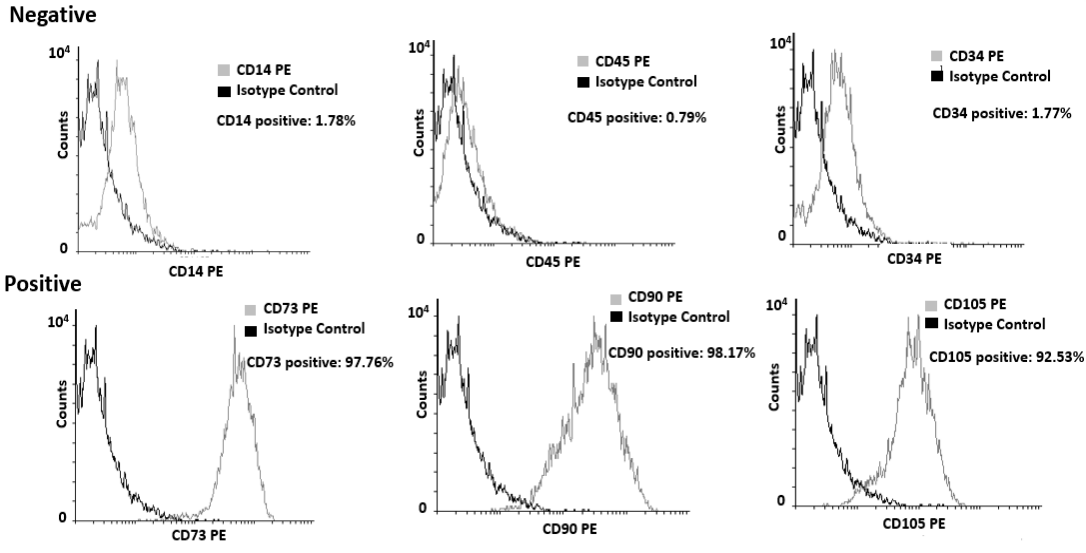
Flow cytometry analysis of cell surface markers presented on hMSCs derived bone marrow

Lack of the hematopoietic markers such as CD34 and CD45 is considered as a minimal immune positive criterion for identification of MSCs while the presence of CD73, CD90, and CD105 are crucial to being positive. The current study illustrated that purified MSCs from human bone marrow were positive for CD73, CD90, CD105 and were negative for CD14, CD34, CD45 (Fig. 1).

Negative: Cell surface markers were quantified by flow cytometry and the positive population of CD14, CD34, and CD45 were notably low with less than 2%. Positive: Crucial positive markers CD73, CD90, and CD105 in isolated bone marrow derived hMSCs were remarkably high with more than 90% of the population.

Exosomes derived from MSC were isolated as described previously. The average size of exosomes was 97.8 nm with the mode of 90 nm measured by Dynamic light scattering (DLS). We examined the MSC exosome morphology by Scanning Electron Microscope (SEM) and the spherical shape of exosomes and the size range of less than 120 nm were confirmed (Data not shown). 

**Figure 2:**
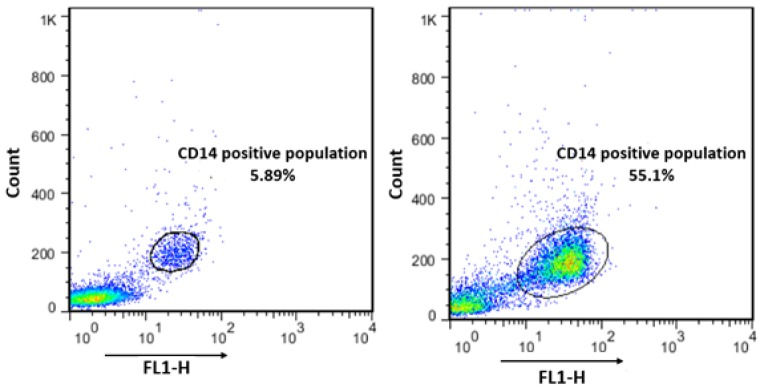
Monocyte Isolation using CD14 negative selection Kit. a) After isolation of PBMCs from whole blood by gradient density centrifuge 5.89% of total cells were CD14. b) After MACS isolation, 55.1% of CD14 positive cells were enriched

In order to confirm the yield and purity of isolated monocytes, after isolation of untouched monocytes, the collected cells were stained by CD14-FITC conjugated antibody. Regarding this, before applying the PBMCs to MACS column, only 5.89% of whole cells were CD14 positive, whereas, after negative selection of CD14 positive cells the purity of CD14 positive cells was reached to 55.1% in collected cells from MACS (Fig. 2).

**Figure 3:**
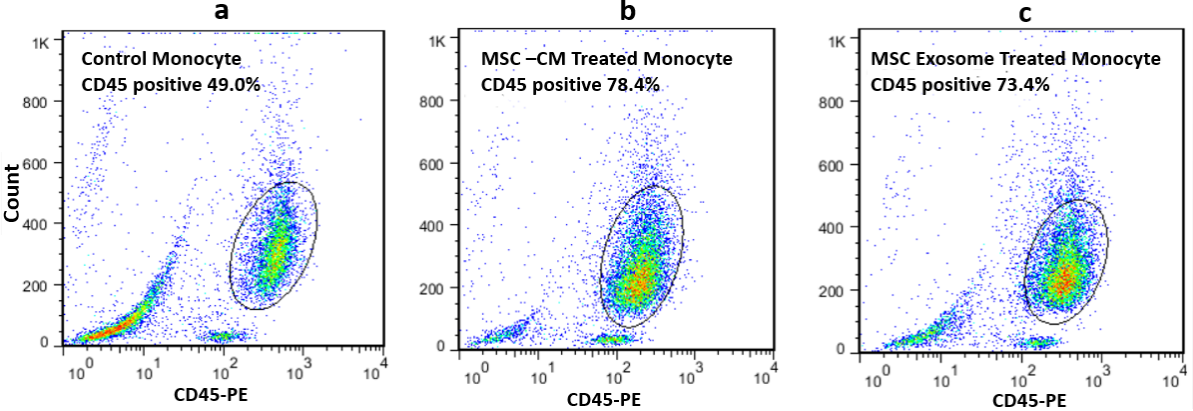
CD45 Flow cytometry analysis. a) After isolation of monocytes, these cells were considered as a control group without any treatment. b) After treatment of monocytes with MSC-CM the increasing number of these population reached to almost 78.4%. Approximately 73.4% of stained cells were CD45 positive after treatment with MSC derived exosome

After isolation, cultivation, and treatment of PBMC-derived monocytes, flow cytometry analysis was performed. Regarding this, CD45 PE-conjugated human monoclonal antibody (anti-CD45) the cellular surface marker of monocytes and macrophages was selected to illustrate the positive population of CD45 with both (MSC-CM) and MSC derived exosome after 72 hours treatment. In the control group, the CD45 positives populations were only 49% before treatment (Fig. 3a). As a result of treatment, the CD45 positive in treating with conditioned media and MSC derived exosomes reached to 78.4% and 73.4% respectively (Fig. 3b,c). However, the identity of differentiated cells after treatment remains obscure and needs more investigation.

**Figure 4:**
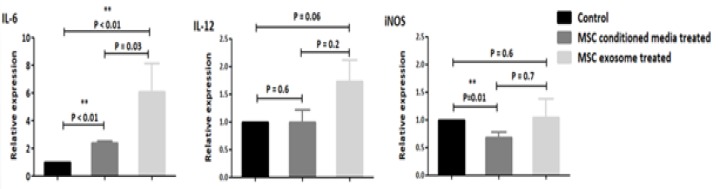
Quantitative RT-PCR results in cultivated monocyte derived from peripheral blood mononuclear cells for pro-inflammatory cytokines after treatment with (MSC-CM) and MSC derived exosomes compared with control group. The data is presented as mean ±SE of three times experiments

MSCs are well known because of their immunoregulatory effect through their secreted factors. The effect of MSC derived exosomes alongside with (MSC-CM) was tested on isolated monocytes from peripheral blood by quantitative PCR- based cytokine gene expression array. The data from Real Time PCR in pro-inflammatory panel illustrated the increased level of IL-6 expression in both exosomes and conditioned media treated cells. Meanwhile, the treated group showed no significant changes in expression of IL-12, moreover, the expression of iNOS in treated cells was declined in conditioned treated as it was hypothesized while exosomes treated group illustrated no meaningful changes (Fig. 4). In anti-inflammatory cytokine panel, alteration in expression of two distinct hallmarks of anti-inflammatory cytokines, IL-10, and EGR-2, were evaluated after treatment. 

**Figure 5:**
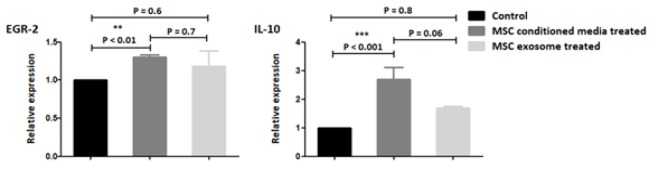
Quantitative RT-PCR results in cultivated monocyte derived from peripheral blood mononuclear cells for anti-inflammatory cytokines after treatment with (MSC-CM) and MSC derived exosomes in contrast with the control group**. **The data is presented as mean±SE of three times experiments

**Figure 6:**
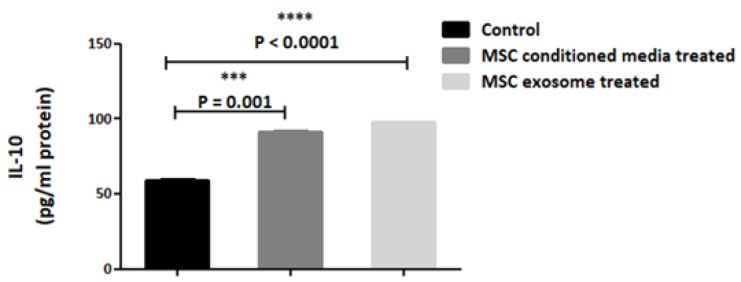
ELISA assay illustrated the elevation of IL-10 protein in collected conditioned media from both treated groups (P<0.0001). In the control group, protein levels were 59.5 pg/ml whereas in MSC-CM and exosome groups were detected 91.8 and 97.9 respectively. The data is presented as mean±SE of three times experiments

In this sense, the conditioned media treated groups exhibited an elevated expression of IL-10 and EGR-2 cytokines, whereas, no significant alteration in their gene expression was detected in exosome treated groups (Fig. 5). IL-10 levels were analyzed by ELISA at 72 hours after treatment. In both (MSC-CM) and MSC exosome treated groups IL-10 levels were markedly increased compared with control group (P<0.0001). Furthermore, the elevation of IL-10 in exosome treated groups exerted more difference in contrast with treatment with (MSC-CM) though this number was not substantial between both treated groups (Fig. 6).

## DISCUSSION

Mesenchymal stem cells have been reputed for their immunoregulatory properties [27]. in immune based disease, including Graft versus Host Disease (GvHD) [28, 29]. type 1 diabetes [30], IBD disease [31] and rheumatoid arthritis [32, 33]. Although many mechanisms underlying these effects are still undefined, many studies have been conducted in the context of MSC therapy in regenerative medicine. MSCs have interaction with arrays of immune cells and that, explains the pro - inflammatory and anti -inflammatory features of MSCs which indicate MSCs are not always immunosuppressive [13 , 34]. Moreover, several studies on animal models of diseases have illustrated the efficacy of conditioned media from MSC cultures [31, 35, 36]. These studies confirmed the consensus on bioactive agents secreted through paracrine mechanisms, which could have beneficial therapeutic effects in different diseases. The latest progress in cell free based therapy is exosomes which are assumed to be present in conditioned media whose play a crucial role in cell-cell communication. 

The components of exosomes and their diversity depend on their cell origin, which these components could be affected by stress or other conditions like cancer or infection [37, 38]. In addition, MSC-derived exosomes have opened the possibility of cell free component utilization, despite that, very little is known about the mechanism underlying exerted modulation effect on various compartments of the immune response [39]. Secreted EVs from MSCs not only induces the proliferation of T cells but also inhibits the natural killer cells [40] and B cells [41].

Interestingly, an abundant number of studies in addition to our presented data have proven that MSCs secrete several soluble factors to promote pro-inflammatory status of monocytes to anti-inflammatory level. These alterations are based on switching their gene expression levels, suggesting the ability of MSC in immune modulation. Moreover, the distinct hallmark of anti-inflammatory cytokine, IL-10, is expressed in the presence of MSCs [42, 43].

Regarding this, in vitro data from our study demonstrated that the secreted pro and anti –inflammatory panel of cytokines measured in culture supernatants is in line with ours and previous studies. Nonetheless there is no report that indicates the immunoregulatory efficiency of MSC-CM and MSC-derived exosomes in comparison with each other, therefore we conducted present experiment. 

The results indicated in pro-inflammatory panel both exosome and MSC-CM elevated the IL-6 gene expression in treated groups where alteration in exosome treated group was substantial. On the other hand, consistent with other reports    [44, 45]  the alteration in IL-12 expression were not significant in neither MSC-CM nor exosome treated monocyte. Alongside with these data, expression of iNOS was declined in MSC-CM treated groups and no alteration occurred in gene expression of exosome treated monocytes. 

Meanwhile, anti-inflammatory cytokine panel, EGR-2, and IL-10 was affected by MSC-CM, while no alteration was observed in exosome treated ones. All these given data, in mRNA levels, indicated that MSC-CM was more effective than MSC derived exosome in alteration of gene expression in monocytes. 

In order to follow up the immunoregulatory efficacy of MSC-CM and MSC derived exosome in protein level, the main hallmark of anti-inflammatory cytokine, IL-10, were assessed by ELISA. Data in this respect revealed that IL-10 protein level was more substantial in exosome treated groups. These findings prove that in the context of MSC-CM and MSC derived exosomes it was the soluble factors to affect the process whose is also confirmed by previous studies [45-47]. Gonzales et al conducted co-culture of colitis derived macrophages with MSC which showed a decline in the secretion of pro- inflammatory cytokines like TNF-α and IL-12 while the level of Il-10 was elevated [48].

In this regard, ample evidence from studies demonstrated that co culture of macrophages with MSCs in the absence of cell-cell contact resulted in M2 phenotypic macrophages secreting the anti-inflammatory cytokines (CD206 ^high^, IL-10 ^high^, IL-12 ^low^) after 48h of culture. Considering this and the result of CD45 marker and changes in gene expression of mentioned cytokines the differentiation of monocytes to M2 macrophages is a possible event. Hence, in the next step of the current study, the effectiveness of MSC-CM and MSC derived exosomes in differentiation of M1 macrophages toward M2 macrophages should be assessed. 

Taken together, these data illustrate the presence of inhibitory and stimulatory factors in MSCs conditioned media and exosomes. According to our obtained findings in vitro, condition media derived from mesenchymal stem cells was more capable in alteration of considered genes and the only exception was IL-6 that was influenced more with MSC derived exosome than condition media. Furthermore, the application of MSC derived exosomes and MSC-CM in the context of immune mediated disorders such as the IBD, GvHD, etc requires more investigation. There is no doubt about the safety and advantages of utilizing MSC-CM and MSC derived exosomes over MSC therapy in immune based disease, though the effectiveness, advantages and disadvantages of either of them should be studied more and for accurate study exosomes and condition media could be characterized separately. 

## References

[1] Pittenger MF, Mackay AM, Beck SC, Jaiswal RK, Douglas R, Mosca JD, Moorman MA, Simonetti DW, Craig S, Marshak DR (1999). Multilineage potential of adult human mesenchymal stem cells. Science.

[2] Wagner W, Wein F, Seckinger A, Frankhauser M, Wirkner U, Krause U, Blake J, Schwager C, Eckstein V, Ansorge W, Ho AD (2005). Comparative characteristics of mesenchymal stem cells from human bone marrow, adipose tissue, and umbilical cord blood. Exp Hematol.

[3] Zuk PA, Zhu M, Ashjian P, De Ugarte DA, Huang JI, Mizuno H, Alfonso ZC, Fraser JK, Benhaim P, Hedrick MH (2002). Human adipose tissue is a source of multipotent stem cells. Mol Biol Cell.

[4] Erices A, Conget P, Minguell JJ (2000). Mesenchymal progenitor cells in human umbilical cord blood. Br J Haematol.

[5] Huang GT, Gronthos S, Shi S (2009). Mesenchymal stem cells derived from dental tissues vs those from other sources: their biology and role in regenerative medicine. J Dent Res.

[6] Gronthos S, Mankani M, Brahim J, Robey PG, Shi S (2000). Postnatal human dental pulp stem cells (DPSCs) in vitro and in vivo. Proc Natl Acad Sci USA.

[7] Ab Kadir R, Zainal Ariffin SH, Megat Abdul Wahab R, Kermani S, Senafi S (2012). Characterization of mononucleated human peripheral blood cells. ScientificWorldJournal.

[8] Somoza RA, Welter JF, Correa D, Caplan AI (2014). Chondrogenic differentiation of mesenchymal Stem Cells: challenges and unfulfilled expectations. Tissue Eng Part B Rev.

[9] Zhang Y, Khan D, Delling J, Tobiasch E (2012). Mechanisms underlying the osteo- and adipo-differentiation of human mesenchymal stem cells. ScientificWorldJournal.

[10] Zeng R, Wang LW, Hu ZB, Guo WT, Wei JS, Lin H, Sun X, Chen LX, Yang LJ (2011). Differentiation of human bone marrow mesenchymal stem cells into neuron-like cells in vitro. Spine.

[11] Aurich H, Sgodda M, Kaltwasser P, Vetter M, Weise A, Liehr T, Brulport M, Hengstler JG, Dollinger MM, Fleig WE, Christ B (2009). Hepatocyte differentiation of mesenchymal stem cells from human adipose tissue in vitro promotes hepatic integration in vivo. Gut.

[12] Le Blanc K, Mougiakakos D (2012). Multipotent mesenchymal stromal cells and the innate immune system. Nat Rev Immunol.

[13] Le Blanc K (2003). Immunomodulatory effects of fetal and adult mesenchymal stem cells. Cytotherapy.

[14] Ciccocioppo R, Bernardo ME, Sgarella A, Maccario R, Avanzini MA, Ubezio C, Minelli A, Alvisi C, Vanoli A, Calliada F, Dionigi P, Perotti C, Locatelli F, Corazza GR (2011). Autologous bone marrow-derived mesenchymal stromal cells in the treatment of fistulising Crohn's disease. Gut.

[15] Duijvestein M, Vos AC, Roelofs H, Wildenberg ME, Wendrich BB, Verspaget HW, Kooy-Winkelaar EM, Koning F, Zwaginga JJ, Fidder HH, Verhaar AP, Fibbe WE, van den Brink GR, Hommes DW (2010). Autologous bone marrow-derived mesenchymal stromal celltreatment for refractory luminal Crohn's disease: results of a phase I study. Gut.

[16] Connick P, Kolappan M, Crawley C, Webber DJ, Patani R, Michell AW, Du MQ, LuanSL, Altmann DR, Thompson AJ, Compston A, Scott MA, Miller DH, Chandran S (2012). Autologous mesenchymal stem cells for the treatment of secondary progressive multiple sclerosis: an open-label phase 2a proof-of-concept study. Lancet Neurol.

[17] Liang J, Zhang H, Hua B, Wang H, Lu L, Shi S, Hou Y, Zeng X, Gilkeson GS, Sun L (2010). Allogenic mesenchymal stem cells transplantation in refractory systemic lupus erythematosus: a pilot clinical study. Ann Rheum Dis.

[18] Sun L, Wang D, Liang J, Zhang H, Feng X, Wang H, Hua B, Liu B, Ye S, Hu X, Xu W, Zeng X, Hou Y, Gilkeson GS, Silver RM, Lu L, Shi S (2010). Umbilical cord mesenchymal stem cell transplantation in severe and refractory systemic lupus erythematosus. Arthritis Rheum.

[19] Bianco P, Robey PG, Simmons PJ (2008). Mesenchymal stem cells: revisiting history,concepts, and assays. Cell Stem Cell.

[20] Karp JM, Leng Teo GS (2009). Mesenchymal stem cell homing: the devil is in the details. Cell Stem Cell.

[21] Akyurekli C, Le Y, Richardson RB, Fergusson D, Tay J, Allan DS (2015). A systematic review of preclinical studies on the therapeutic potential of mesenchymal stromal cell-derived microvesicles. Stem Cell Rev.

[22] Robbins PD, Morelli AE (2014). Regulation of immune responses by extracellular vesicles. Nat Rev Immunol.

[23] Zhang B, Yeo RW, Tan KH, Lim SK (2016). Focus on Extracellular Vesicles: Therapeutic Potential of Stem Cell-Derived Extracellular Vesicles. Int J Mol Sci.

[24] Mantovani A, Sica A, Sozzani S, Allavena P, Vecchi A, Locati M (2004). The chemokine system in diverse forms of macrophage activation and polarization. Trends Immunol.

[25] Gordon S, Taylor PR (2005). Monocyte and macrophage heterogeneity. Nat Rev Immunol.

[26] Baghaei K, Hashemi SM, Tokhanbigli S, Asadi Rad A, Assadzadeh-Aghdaei H, Sharifian A, Zali MR (2017). Isolation, differentiation, and characterization of mesenchymal stem cells from human bone marrow. Gastroenterol Hepatol Bed Bench.

[27] Le Blanc K, Tammik C, Rosendahl K, Zetterberg E, Ringdén O (2003). HLA expression and immunologic properties of differentiated and undifferentiated mesenchymal stem cells. Exp Hematol.

[28] Le Blanc K, Rasmusson I, Sundberg B, Götherström C, Hassan M, Uzunel M, Ringdén O (2004). Treatment of severe acute graft-versus-host disease with third party haploidentical mesenchymal stem cells. Lancet.

[29] Le Blanc K, Frassoni F, Ball L, Locatelli F, Roelofs H, Lewis I, Lanino E, Sundberg B, Bernardo ME, Remberger M, Dini G, Egeler RM, Bacigalupo A, Fibbe W, Ringdén O; Developmental Committee of the European Group for Blood, Marrow Transplantation (2008). Mesenchymal stem cells for treatment of steroid-resistant, severe, acute graft-versus-host disease: a phase II study. Lancet.

[30] Madec AM, Mallone R, Afonso G, Abou Mrad E, Mesnier A, Eljaafari A, Thivolet C (2009). Mesenchymal stem cells protect NOD mice from diabetes by inducing regulatory T cells. Diabetologia.

[31] Pouya S, Heidari M, Baghaei K, Asadzadeh-Aghdaei H, Moradi A, Namaki S, Zali MR, Hashemi SM (2018). Study the effects of mesenchymal stem cell conditioned medium injection in mouse model of acute colitis. Int Immunopharmacol.

[32] Wang L, Cong X, Liu G, Zhou J, Bai B, Li Y, Bai W, Li M, Ji H, Zhu D, Wu M, Liu Y (2013). Human umbilical cord mesenchymal stem cell therapy for patients with active rheumatoid arthritis: safety and efficacy. Stem Cells Dev.

[33] El-denshary ESM, Rashed LA, Elhussiny M (2012). Mesenchymal stromal cells versus betamethasone can dampen disease activity in the collagen arthritis mouse model. Clin Exp Pharmacol.

[34] Nauta AJ, Fibbe WE (2007). Immunomodulatory properties of mesenchymal stromal cells. Blood..

[35] Wang W, Du Z, Yan J, Ma D, Shi M, Zhang M, Peng C, Li H (2014). Mesenchymal stem cells promote liver regeneration and prolong survival in small-for-size liver grafts: Involvement of C-Jun N-terminal kinase, cyclin D1, and NF-kB. PLoS One.

[36] Ionescu L, Byrne RN, van Haaften T, Vadivel A, Alphonse RS, Rey-Parra GJ, Weissmann G, Hall A, Eaton F, Thébaud B (2012). Stem cell conditioned medium improves acute lung injury in mice: in vivo evidence for stem cell paracrine action. Am J Physiol Lung Cell Mol Physiol.

[37] Roccaro AM, Sacco A, Maiso P, Azab AK, Tai YT, Reagan M, Azab F, Flores LM, Campigotto F, Weller E, Anderson KC, Scadden DT, Ghobrial IM (2013). BM mesenchymalstromal cell-derived exosomes facilitate multiple myeloma progression. J Clin Invest.

[38] Im H, Shao H, Park YI, Peterson VM, Castro CM, Weissleder R, Lee H (2014). Label-free detection and molecular profiling of exosomes with a nano-plasmonic sensor. Nat Biotechnol.

[39] Théry C, Amigorena S, Raposo G, Clayton A (2006). Isolation and characterization of exosomes from cell culture supernatants and biological fluids. Curr Protoc Cell Biol.

[40] Liu C, Yu S, Zinn K, Wang J, Zhang L, Jia Y, Kappes JC, Barnes S, Kimberly RP, Grizzle WE, Zhang HG (2006). Murine mammary carcinoma exosomes promote tumor growth by suppression of NK cell function. J Immunol.

[41] Köppler B, Cohen C, Schlöndorff D, Mack M (2006). Differential mechanisms of microparticle transfer toB cells and monocytes: anti-inflammatory propertiesof microparticles. Eur J Immunol.

[42] Zhang QZ, Su WR, Shi SH, Wilder-Smith P, Xiang AP, Wong A, Nguyen AL, Kwon CW, Le AD (2010). Human gingiva-derived mesenchymal stem cells elicit polarization of m2 macrophages and enhance cutaneous wound healing. Stem Cells.

[43] Barminko J, Kim JH, Otsuka S, Gray A, Schloss R, Grumet M, Yarmush ML (2011). Encapsulated mesenchymal stromal cells for in vivo transplantation. Biotechnol Bioeng.

[44] Vasandan AB, Jahnavi S, Shashank C, Prasad P, Kumar A, Jyothi Prasanna S (2016). Human Mesenchymal stem cells program macrophage plasticity by altering their metabolic status via a PGE 2-dependent mechanism. Sci Rep.

[45] Kim J, Hematti P (2009). Mesenchymal stem cell-educated macrophages: a novel type of alternatively activated macrophages. Exp Hematol.

[46] Yu X, Wu B, Ma T, Lin Y, Cheng F, Xiong H, Xie C, Liu C, Wang Q, Li Z, Tu Z (2016). Overexpression of IL-12 reverses the phenotype and function of M2 macrophages to M1 macrophages. Int J Clin Exp Pathol,.

[47] Brundu SFA (2015). Polarization and Repolarization of Macrophages. J Immunol.

[48] González MA, Gonzalez-Rey E, Rico L, Büscher D, Delgado M (2009). Adipose-derived mesenchymal stem cells alleviate experimental colitis by inhibiting inflammatory and autoimmune responses. Gastroenterology.

